# Acceptability and impact of group interpersonal therapy (IPT-G) on Kenyan adolescent mothers living with human immunodeficiency virus (HIV): a qualitative analysis

**DOI:** 10.1186/s12905-022-01807-w

**Published:** 2022-06-18

**Authors:** Obadia Yator, Lincoln Khasakhala, Grace-John Stewart, Manasi Kumar

**Affiliations:** 1 Department of Psychiatry, School of Medicine, College of Health Sciences, University of Nairobi, P. O. Box 799-00517, Nairobi, Kenya; 2 Department of Psychiatry, University of Nairobi, P. O. Box 30197-00100, Nairobi, Kenya; 3 University of Washington, 325 9th Avenue, Box 359909, Seattle, WA 98104 USA; 4 Department of Psychiatry, College of Health Sciences, University of Nairobi, P.O. Box 47074-00100, Nairobi, Kenya; 5 Department of Psychology, University College London, London, UK

**Keywords:** Task shifting, Postpartum depression, HIV-related stigma, Group Interpersonal Psychotherapy, Community health volunteers

## Abstract

**Background:**

Task shifting is a well-tested implementation strategy within low- and middle-income countries that addresses the shortage of trained mental health personnel. Task shifting can increase access to care for patients with mental illnesses. In Kenya, community health workers (CHWs) are a combination of community health assistants and community health volunteers and have played a crucial role on this front. In our study, we seek to assess the acceptability and feasibility of Group Interpersonal Psychotherapy (IPT-G) delivered by CHWs among depressed postpartum adolescents (PPAs) living with human immunodeficiency virus (HIV).

**Method:**

The study used theoretical framework of behaviour change including: Capability, Opportunity and Motivation (COM-B model) to help understand behavioural changes due to IPT-G intervention delivered by the CHWs. 24 PPAs were administered IPT-G by trained CHWs from two health centres. A two-arm study design (IPT-G intervention and treatment as usual) with an intent to treat was used to assess the acceptability and feasibility of IPT-G. With purposeful sampling, participants who scored > 10 on the Edinburgh postnatal depression scale and who were 6–12 weeks postpartum were eligible for the study. Participants were equally distributed into two groups: one group for intervention and another as a wait-listed group. This was achieved by randomly allocating numerical numbers and separating those with odd numbers (intervention group) and even numbers (wait-listed group). Focus group discussions and in-depth interviews ascertained the experiences and perceptions of the PPAs and the CHWs during IP-G delivery process. In addition to weekly face-to-face continuous supportive supervision for the CHWs, the researchers also utilized phone calls, short messages services and WhatsApp instant messaging services.

**Results:**

The CHWs found the intervention useful for their own knowledge and skill-set. With regards to participation, 21 out of the 24 adolescents attended all sessions. Most of the adolescents reported an improvement in their interpersonal relationships with reduced distress and lessening of HIV-related stigma. Primary healthcare workers embraced the intervention by accommodating the sessions in their routine clinic activities.

**Conclusion:**

Our study demonstrates the possible benefits of task shifting in addressing mental health problems within low-resource settings in Kenya, and IPT-G is demonstrated to be both acceptable and feasible by health workers and adolescents receiving care.

**Supplementary Information:**

The online version contains supplementary material available at 10.1186/s12905-022-01807-w.

## Background

### Shortage of mental health personnel and associated challenges

The chronic shortage of well-trained health personnel in Sub-Saharan Africa (SSA) necessitated task shifting as a key implementation strategy. Task shifting in SSA health systems typically means that the CHWs are trained to help increase the number of health services being provided to reduce cost and improve the delivery of care [1]. The concept of task shifting involves rational distribution from well-trained to less specialized health workers or those who have been trained on a limited time duration on specific skills in a given area of need [2]. Several barriers to task shifting revolve around the need to strengthen health systems by improving systemic and physical structures [3]. The CHWs undergo challenging experiences while task shifting: undertaking tasks that may drain them emotionally and physically, persistent problems of inadequate training, unstructured supervision, and poor remuneration or complete lack of reward or entitlements to any form of benefit in some instances [4].

Task shifting is meant to reduce workloads for overburdened specialist health workers and improve patients’ linkage to services [5]. In Kenya, the CHWs are not well compensated and sometimes expected to work on a voluntary basis with poorly structured responsibilities causing them to take roles requiring more skills than their abilities [6]. Embracing task shifting of key preventive and promotive activities in the HIV programme using the CHWs promises to be a good step towards achieving the 90-90-90 goals. UNAIDS has in the past identified the engagement of community workers as essential in HIV prevention and advocacy [7].

### Prevalence of HIV in peripartum adolescents in Kenya

Our study targets one of the most vulnerable youth populations: adolescent mothers living with HIV. In SSA, adolescent girls aged 15 to 24 years represent 10% of the population, and using 2017 estimates, this group accounts for 25% of the new HIV infections [8]. The prevalence of HIV in Kenya among female adolescents aged 15–24 is 4% [9], and these young girls are two times more likely to contract HIV than their male counterparts [10].

The overall prevalence of adolescent pregnancy in Africa is 18.8% and 19.3% for the Sub-Saharan region [11]. By the age of 18, 42% of adolescents from SSA living in urban areas will have become pregnant, and more than 50% of their rural counterparts would be so too [12]. Such early pregnancies also increase their chances of HIV infections [13]. Most adolescents are seen to be infected with HIV by older men, aged late 20 s and early 30 s, who may not even be aware of their status, and thus, unlikely to be on anti-retroviral therapy (ART) [14].

A study in Malawi found that adolescents living with HIV had a depression prevalence of 18.9% [15]. A similar study in Kenya focusing on mental health outcomes among adolescents living with HIV documented almost the same depression prevalence of 17.8% [16]. Worldwide, postpartum depression (PPD) prevalence in adolescents is higher than that for adults, and it is estimated to range from 26 to 50% [17–20].

### The role of psychosocial interventions for adolescents

Psychological interventions have been recommended for persons living with HIV to mitigate common mental health illnesses, including depressive illnesses [21], with no side effects against ART [22]. Psychological interventions for adolescents should aim at addressing issues related to psychosocial development, training on social skills including life skills, and shaping their behaviours towards a productive future life through livelihood and vocational training [23]. It has been found that adolescents have significant risks of frequent unprotected sex with an equally high risk of contracting HIV and having an unplanned pregnancy or both at some time in their lives [24]. Adolescents engage in risk-taking behaviours and girls may be less assertive to negotiate for condoms. Hence, higher chances of unprotected sex are serious considerations [25] in trying to address the well-being of adolescent girls.

Prevention of mother-to-child transmission (PMTCT) clinics have provided a conducive environment for addressing the life challenges associated with an HIV infection among perinatal women. In the year 2017, the global ART coverage among men aged 15 years and above was 53% compared with 65% of women of the same age [8]. In a recent study from six sub-Saharan countries, HIV-related stigma has been associated with delays in treatment and difficulties in adhering to ART, as shown in some systematic review studies [26, 27]. A noteworthy emerging finding is that depression treatment improves adherence to ART, which is key to an improved quality of life [28, 29].

A Nigerian study on psychological intervention for adolescents living with HIV utilized support groups using Facebook groups for a five-weekly session. The ability for adolescents to interact, learn more about HIV, share experiences and their fears on social media was seen to help them in coping with their status [30].

### Group Interpersonal Psychotherapy (IPT-G) and its relevance for this vulnerable population

A study assessed interpersonal relationships between the youth and their families and found out that poor relationships led to depression [31]. Lower caregiver supervision was also associated with higher depression [32]. IPT-G is well-poised to help address interpersonal difficulties that adolescents are predisposed to. It is conceptualized around four problem areas: *grief and loss*, *interpersonal role disputes*, *role transitions*, *and interpersonal deficits/social isolation*. During the adaptation of IPT-G for depressed adolescents, Mufson et al. [33] reported that the intervention targets an individual’s interpersonal relationships with other persons in a given family/society where therapeutic benefits are achieved.

For example, when PPAs living with HIV are put together in a therapy session, they appreciate that their unpleasant experiences in life are also experienced by other persons in similar situations. This motivates the adolescent to try new interpersonal interactions that will enhance better social functioning in society. IPT-G provides adolescents with peers who have similar difficulties and utilizes synergies in groups to understand their interpersonal problems and develop new ways of coping.

Our study is in line with a call by the World Health Organization (WHO) to embrace the strengthening of universal health coverage. Primary health care depends on health system structures acknowledging various levels of health care services where specialized health workers are deployed at the referral levels, and the CHWs are based at the community level and both are interdependent [34–36]. We aim to assess qualitatively the acceptability and feasibility of IPT-G for PPAs living with HIV being delivered by the trained and supervised CHWs within routine clinical settings in Nairobi, Kenya.

### Theoretical framework

**Fig. 1:**
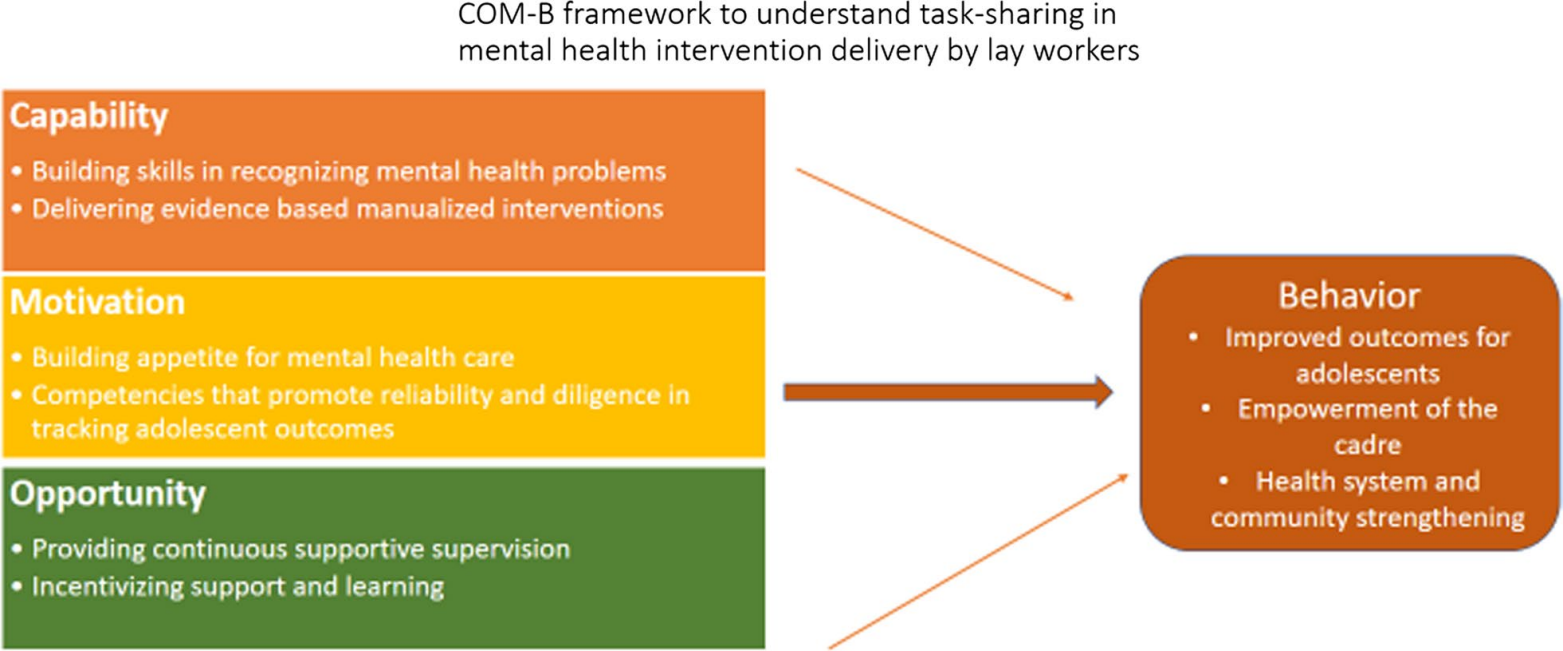
Theorethical framework

Our study adopted a theoretical framework of behaviour change which included: Capability, Opportunity and Motivation (COM-B model) to help understand behavioural changes due to IPT-G intervention being delivered by the CHWs to Kenyan adolescent mothers living with HIV [37]. The acceptability and impact of IPT-G when delivered by lay workers were identified by using this framework (see Fig. 1). In this framework, capability (building skills on IPT-G), motivation (competencies in IPT-G) and opportunity (provision of continuous supportive supervision) will result in the enactment of a particular kind of behavior (improved outcomes for adolescents, and improved health system and community strengthening).

## Method

### Study design, participants, setting and approvals

Our study aimed to assess the acceptability and feasibility of IPT-G delivered by CHWs among depressed PPAs living with HIV in routine clinical settings. We adopted a theoretical framework of behaviour change (COM-B model) to help understand how CHWs can deliver IPT-G. The findings of this qualitative study will inform the modalities of task shifting, where IPT-G could be delivered by the CHWs under continuous supportive supervision to compensate for the shortage of trained mental health specialists within Kenya.

We conducted our intervention between August 2018 and July 2019. Our pilot trial was a mixed methods study, and for this article, our focus is on the qualitative findings to highlight the acceptability and feasibility of IPT-G when delivered by trained non-medical specialists. The CHWs included CHAs (n = 2) and CHVs (n = 6) who delivered IPT-G to the PPAs. Both study sites had the same numbers of CHWs, with all participants signing a written informed consent. 24 PPAs aged 15–24 years old and living with HIV were recruited from two sites: the Kangemi health centre and the Kariobangi health centre. They were also expected to be 6–12 weeks postpartum. The two study sites were chosen since they are situated on two opposite poles of Nairobi city, and both are home to a population of low economic status.

Ethical approval was attained from the Kenyatta National Hospital-University of Nairobi Ethics and Research Committee (Approval No. P97/02/2018). Thereafter, the Nairobi county health services were informed of the intent to conduct the study. The health enter staff were all ready to support the study since it was understood to be targeting a specific population known to have myriad psychosocial issues. The data that support the findings of this study are available with the corresponding author on request.

### Training and supervision of the CHWs

The CHWs undertook a two-day training on IPT-G with extensive sessions of role-plays. Six (6) PPAs who scored above 10 on the EPDS were recruited for piloting IPT-G. Continuous supportive supervision to the CHWs was undertaken by the lead researcher, clinical supervisor who was a final year student pursuing Masters of Science in Clinical Psychology, and a research assistant who helped oversee the recruitment of the participants. A WhatsApp group for the CHWs was formed to ease communication and coordination during the study period.

### Sampling and recruitment of participants

The participants who had EPDS scores > 10 were eligible for the study. A total of forty-six [46] PPAs were screened at the PMTCT clinic, and thirty-two (32) of them fulfilled the inclusion criteria, of which twenty-five (25) consented to receive the intervention. The PPAs were randomly allocated numerical numbers from 0 to 25, and after that, odd numbers were picked for the intervention group (n = 13), and even numbers were picked for the wait-list group (n = 12). One participant from the intervention group dropped out at the initiation of IPT-G, leaving 24 to proceed to complete the study. The intervention group initiated IPT-G and completed it within 8 weeks, and thereafter the wait-list group also received IPT-G. The CHWs delivered IPT-G to the two groups weekly for eight sessions as per the WHO protocol [38].

For our qualitative findings, purposeful sampling was used after the intervention to recruit PPAs (n = 19) and CHWs (n = 7) for FGDs to allow them to share their experiences and perceptions about IPT-G. The FGDs were conducted among the CHWs (2 pre-trial within each respective study sites and 1 post-trial with a combination of all the CHWs). Three audio recordings from the eight IPT-G sessions were randomly selected from each study site to inform us on their experiences and observations. In-depth interviews were conducted among the health care workers within the two sites: nursing officer-in-charge (n = 2), mentor-mother (n = 2), laboratory technologist (n = 2), PMTCT-nurse (n = 2) and CHAs (n = 2).

### Data collection tools

Separate FGDs among the PPAs and the CHWs were conducted after IPT-G intervention. In-depth interviews for the medical staff and the CHAs within the two study sites were also conducted. Screening for depressive symptoms was carried out using the EPDS [39], to monitor the change in scores following IPT-G intervention. Interpersonal inventory was used to assess the interpersonal relations (emotional attachments and social interaction) among the PPAs. All the participants (PPAs, CHWs and health care workers) were acquainted with both English and Kiswahili, hence they were comfortable with communication during the discussions and interviews.

The audio was recorded for all sessions to enable the auditing of each weekly session as they progress to improve therapy quality. Weekly field notes for the 16 sessions were kept to document observations, experiences and perceptions of the CHWs, the PPAs and health workers within the facility (see Table 1).

**Table 1:** Sociodemographic characteristics of the respondents

Variable	Category	Overall (N = 24)	Intervention (N = 12)	Waitlist (n = 12)
Age	18–20 Years	3 (12.5%)	2 (66.7%)	1 (33.3%)
	21–24 Years	21 (87.5%)	10 (47.6%)	11 (52.4%)
Marital status	Without a partner	7 (29.2%)	4 (57.1%)	3 (42.9%)
	With a partner	17 (70.8%)	8 (47.1%)	9 (52.9%)
Education level	Primary and below	11 (45.8%)	4 (36.4%)	7 (63.6%)
	Secondary and above	13 (54.2%)	8 (61.5%)	5 (38.5%)
Occupation	Employed	5 (20.8%)	4 (80.0%)	1 (20.0%)
	Unemployed	19 (79.2%)	8 (42.1%)	11 (57.9%)
Income per month (Kenya shillings)	0–10,000	22 (91.7%)	11 (50.0%)	11 (50.0%)
	10,001–20,000	2 (8.3%)	1 (50.0%)	1 (50.0%)
Number of children	1	11 (45.8%)	5 (45.5%)	6 (54.5%)
	2	8 (33.3%)	5 (62.5%)	3 (37.5%)
	3	3 (12.5%)	1 (33.3%)	2 (66.7%)
	4	2 (8.3%)	1 (50.0%)	1 (50.0%)
Reaction to HIV status	Accepted	13 (54.2%)	9 (69.2%)	4 (30.8%)
	Not accepted	11 (45.8%)	3 (27.3%)	8 (72.7%)
HIV status of child	Negative	10 (41.7%)	5 (50.0%)	5 (50.0%)
	Not sure	14 (58.3%)	7 (50.0%)	7 (50.0%)
Intimate partner Violence	Yes	13 (54.2%)	6 (46.2%)	7 (53.8%)
	No	11 (45.8%)	6 (54.5%)	5 (45.5%)

### Data analytic approach

Transcription of audio-recorded qualitative data and field notes were coded to identify common emerging themes, highlighting the barriers and facilitators of IPT-G. Three (3) coders participated in the process whereby the lead researcher, clinical supervisor, and research assistant participated in coding and thereafter the three mentors of this study looked at the overall themes generated from the study [40]. Finally, we determined feasibility by evaluating the recruitment process, weekly observations during sessions and completion rate of the intervention session.Acceptability was determined by inquiring how individual participants felt about the intervention, participant’s knowledge, perceptions, barriers, and gains achieved from the intervention [41] (see Table 2).

**Table 2:** Data collection instruments

Constructs/and domains	Activities	Parameters
IPT inventory	Assess quality of Interpersonal relationship for postpartum adolescents	Weekly questionnaire to assess interaction changes
Knowledge of IPT and delivery skills	Make field notes during session which records: (i) Depression symptoms for each participant (ii) Progress in IPT problem areas (iii) Plan for next session If the participant fails to attend session or dropped out, the CHWs should state the reason and plans for contacting her	CHWs weekly notes during session (Filled by the CHW)
IPT process monitoring	Clinical supervisor issues printed copies on protocol to CHWs to guide them on tasks, steps and techniques to be used from pre-group phase, initial phase (session 1), middle phase (session 2–7) and termination phase (session 8)	Session-by-session IPT activities (Issued by clinical supervisor to CHWs)
	Competency: CHW’s level of IPT-G understanding from the training, piloting, supervision and mentorship. Clinical supervisor reports the strengths, difficulties and prescribe plans for improvement	Pre-group phase (Clinical supervisor asses individual CHW on their ability to administer IPT-G)
Feasibility and acceptability	Clinical supervisor and research assistant conducted FGDs for CHWs prior and after IPT-G intervention. FGDs was also conducted for PPAs after intervention at the 4 months of follow-up. In-depth interviews for: Nursing officer in-charge, Laboratory technologist, PMTCT-Nurse, CHA, and Mentor -mother at the two study sites	FGDs, Key Informants interviews, and observations

## Results

The CHWs who delivered IPT-G under continuous supportive supervision included two (2) CHAs and six (6) CHVs from each study site. The CHVs had a combined average age of 37 years and 9 months and the CHAs were women in their thirties. The distribution of education level for the CHWs was as follows: post-secondary school (n = 4), grade 12 (n = 1), grade 10 (n = 1), and below grade 8 (n = 2). Our IPT-G intervention being delivered by the CHWs helped adolescents by improving their interpersonal relationships, communication processes and overall mental health, which affirms the positive impact it has through task shifting within routine clinical settings.

Among the 24 PPAs who received IPT-G, the majority (21, 87.5%) were aged between 21 and 24 years old, and were mostly residing with their partners (17, 70.8%) with parity of fewer than 2 children (20, 83.3%). About half had attained secondary and above education levels (despite unemployment ranking high at 79.2%), and with most of them earning less than 100 USD per month (22, 91.7%) (see Table 1).

The study had a retention and follow-up rate of 21 (87.5%) out of the 24 participants. Among the 8 CHWs recruited to deliver IPT-G, one of the CHVs unfortunately suddenly passed away, and thus the remaining 7 CHWs participated to completion of the intervention. All the CHWs were in concurrence that the IPT-G delivery process was workable considering it was held weekly and hence that sort of time allocation was possible.

### Qualitative acceptability and feasibility data

The IPT-G as an evidenced-based psychological intervention was found to be feasible and acceptable as per the narrations of the PPAs and the CHWs during the FGDs, and also in-depth-interviews from the facility health workers. Trained and supervised CHWs were able to adhere to the IPT-G protocol, and the context of primary healthcare was seen to be good. We observed that, IPT-G among the PPAs living HIV improved their communication, interpersonal skills building, anger management, instilled hope despite their HIV status, empowered them within the community in terms of job placements and enhanced cohesion among these vulnerable population at the health facility and community level.

CHWs was found to be important players towards the implementation of IPT-G since it is an intervention with easy to follow protocol. CHWs act as a link between the health facility and community, provide follow-up to participants up to their residence within the community.With their limited skills on specialized health services, CHWs are not pre-occupied at the health facility.

We structured our findings by highlighting the acceptability and impact of IPT-G when delivered by lay workers into three domains towards enacting behavioural change (improved outcomes for adolescents, improved health system and community strengthening) after the intervention: (1) capability-building skills on IPT-G, (2) motivation-competencies in IPT-G, and (3) opportunity-provision of continuous supportive supervision.

### Capability

The CHWs found the intervention useful in terms of how the intervention built their knowledge and skills and successfully delivered IPT-G. During the training of the lay health workers, they demonstrated a good understanding of depression and felt competent to deliver IPT-G. The adolescent mothers benefited from IPT-G as they narrated how they could now function better, communicate better with their families and partners, interact socially, manage their anger and even resume work for an income or education (see Table 3).

**Table 3:** Ideas that appealed to the adolescents

IPT-G informed themes	Relevant quotations
Improved communication	*“I can say that we learnt how to communicate, communication.” (MM, age 24, Kangemi)* *"I had an issue with my parents, but now we are okay, and we communicate well". (RJ, age 23, Kangemi)* *“About my estranged partner, I can say that at the moment, even though we have not yet met, we are fine because we are communicating.” (MM, age 24, Kangemi)* *“This group has helped me in terms of communication.” (JF, age 24, Kangemi)* *“It helped me to interact with people.” (RA, age 21, Kangemi)*
Improved anger management	*"I am called S, but that one is informal, but in reality, I am called NC; by the way, even me, it has helped me so much, I have seen that several things have changed. The anger I used to have is no longer there; I just feel that I am okay.” (NC, age 24, Kariobangi)* *"For me, I got assistance because even now I see that getting angered is not so much there, I just see that life is okay; I don't want- I mean I know how to control anger, and it cannot rise the way it used to happen to me.” (JA, age 22, Kariobangi)*
IPT-G delivery process	*“They were nice on my side, caring, they used to concentrate on us, they were social; let me say they were just nice.” (JF, age 24, Kangemi)* *“They used to understand us.” (JI, age 21, Kangemi)* *“They were nice people; in case you got stuck on something they could help you [Inaudible].” (SK, age 23, Kangemi)* *“They used simple terms….and for those that were difficult they used to elaborate.” (MR, age 24, Kangemi)* *“They were just using simple terms.” (RA, age18, Kangemi)*
Overall Perceptions towards IPT by postpartum adolescents	*“I can tell him(lead researcher) thanks because he has helped us so much, we had stress, we were lonely but the way he organized this group it has helped us and we have found means of helping our colleagues out there and that he should just continue that way without giving up and God grant him strength and life.” (FW, age 21, Kariobangi)* *"I can tell him (lead researcher) that he assisted us so much because if some of us could still be where we used to be then, we wouldn't exist till now; but he did an important thing and assisted us so he should continue that way and may God bless him.” (NC, age 24, Kariobangi)* *"I can tell him (lead researcher) thanks for creating this organization it has helped all of us on how we can also educate other people to be happy, and he should just continue with that spirit.” (LA, age19, Kariobangi)* *"I can tell him thanks so much, he helped us a great deal because we came here with stress, but we have been assisted, and we say thank you so much and it should just continue this way [Crosstalk].” (SA, age 23, Kariobangi)* *"I want to tell him (lead researcher) thanks for he has made me able to believe in myself, and may God give him strength to proceed with this program for others who are behind and are like us to get assistance.” (EA, age 24, Kariobangi)* *"For me, it had helped me, I have lived positively and I am not bothered by what people say [Some silence].” (LA, age 19, Kariobangi)* *“I am EAO and for me this content made me more aggressive, I mean I am not the way I used to be; I have many differences such that when I walk in other places I am not afraid. I know who I am now, I have decided to live a positive life and I am used to it, and if I see someone else I teach her the way I have been taught.” (EO, age 24, Kariobangi)*

The CHWs were very satisfied with their achievements and some shared their experiences on the milestones towards IPT-G delivery:

*At the beginning of the sessions, there were challenges because all the people were still new, so it is later that people came to know each other and developed trust in the group and everyone could say all her issues* (SK, age 50, CHV, Kariobangi).

One of the CHWs underscored the potential impact IPT-G in the community as indicated in her explanation:

… *it has changed us, as much as we were the teachers, but we feel that it helped us. So, I just pray that more of that kind comes and the way I said that when you just change one person, he/she will change five others and those five others will change others, and at the end of the day you may find that you have changed the whole country and even the world, so thank you* (JA, age 24, CHV, Kangemi).

One of the CHAs affirmed that the time for IPT-G sessions could easily embedded into their routine activities since it consumed only 90 min once a week.

*Not a burden because it was usually once a week, and if you are meeting those clients later, it is the one in which you can negotiate the time that you will meet, so I don’t think, maybe if others think otherwise* (LN, age 34, CHA, Kangemi).

After our study, most health care providers at the two health centres appreciated the impact of IPT-G on adolescents attending the PMTCT clinic, citing improved social functioning, better communication and appealing personal hygiene/grooming by the adolescent mothers. As an illustration to share this point further, one of our participants was grateful for participating in the sessions and shared her positivity:

*I can remember there is one who said after the sessions,* ‘*nowadays I can get out and talk with women, I can go out of the gate because when I was alone, I felt that I am not okay because I have HIV and I have given birth at a younger age. But now I have gotten that courage after these sessions to go outside I can talk, I have that courage,*’ *so that made her feel that they are many and she is not alone*” (LN, age 34, CHA, Kangemi).

“*It was just like Kangemi; at first, for the people with whom you are not familiar enough, they could not open up; you ask her a question, and she feels like where do you want to lock and take her? But now the second time, they will be free, and the third time she will feel like* ‘*I can remember what happened to me and how it is on somebody else,*’ *because we had supervision like from [the] CHA and MK and could correct us when [we were] wrong and it becomes normal*” (SN, age 65, CHV, Kariobangi).

“*I think even these girls have become role models in the community … we told them* ‘*if you find somebody who is in a situation maybe you can assist and feel that it [is] closer to yours or it is similar to yours, you are now empowered, and you can help this person at your level and if you feel that it is difficult is when you can refer.*’ *Still, now they are doing on their own, which is good* (EO, age 24, CHV, Kariobangi).

### Motivation

The intervention empowered some of the adolescent mothers to help others, which was one of our intended purposes towards disseminating benefits to the community level:

*Even the sessions were very good; because some used to come and tell you, I have this and that problem and I passed through this, but I went and did what you told me, and I have succeeded, you get it? One member in our group, I recall, and even at the moment, she normally rings me and tells me,* ‘*that thing helped me, and I am able to help others*’ (NG, age 43, CHV, Kangemi).

The IPT-G sessions formed a part of the entry point to enlarging the scope of their social support and even after the intervention they continued to share their issues:

*So, it was a very big problem at the beginning, but I am glad that as the sessions were proceeding, we became good friends; actually, we developed a rapport, and even until now, some still call* ‘*when are we meeting again?*’ *[Laughter]*… (EO, age 24, CHV, Kariobangi).

Others were of the view that follow-up is important because most of the participants open up to their issues in the middle of the sessions:

*What I can say; when I joined—let me talk about group two, we came well but after attending like three sessions is when you could see them now pouring out [Laughter]* … *they tell you all their troubles in life*… (FC, age 30, CHV, Kangemi).

The CHWs were now able to connect easily with the adolescent mothers after bonding with them during the sessions:

*So, we are also still doing follow-ups. Some even call by themselves, sometimes I call them, some even when they come for clinics, they just come looking for us which is good so, so they trust us. So, there is that trust, there is that friendship, there are so many benefits that came out of this, so I was just gladly sharing* (EO, age 24, CHV, Kariobangi).

Our adolescent participants acknowledged that IPT-G helped alleviate social isolation, anger, hopelessness and low mood which are typical depressive symptoms. After IPT-G intervention, our adolescent participants now acknowledged that they lived with horrible thoughts and a feeling of hopelessness about their past and felt liberated and at great ease with their new situation. They narrated how they were able to socialize with others and perform their family responsibilities effectively including looking after the baby without negative self-perceived stigma despite them living with HIV (see Table 4).

*I am called JI, and it helped me now, I can talk to people, I was hopeless but now…. yes, I am of importance. When I saw that I am this way, I felt that there is no life and that I am not important* (JI, age 21, Kangemi).

One of the adolescent mothers narrated how HIV-related stigma used to torment her before our intervention:

*I know myself since I know how I used to feel; when I sit down, I ask myself,* ‘*what kind of life is this?*’ *I used to feel guilty. I don’t want people’s stories. I just feel that a story may arise and reach the point of the infection, so what will I say? You know at that point you will just be forced to remain silent; you will not talk—you will never have what to say because they are negative, and you are positive, and they want to talk about that infection* … (JA, age 22, Kariobangi).

It was clear that irritability was negatively affecting communication with their partners as one of the participants narrated how she used to live while having depressive symptoms:

*In the past, whenever I could get angry, I could not cook…. I cannot eat, if it is talking to people, I cannot talk to him; everybody sits apart when it comes to washing, everybody washes their clothes* (SK, age 23, Kangemi).

**Table 4:** Postpartum adolescents’ understanding of IPT-G intervention

IPT theme	Quotations
Overall impact of IPT (Reduce social isolation, anger, hopelessness, low mood)	*“I used to stay in the house asking myself 'who am I for sure? Whose friend can I be?' ‘so, I feel like even taking poison, I think of many things; but I have seen a great difference; when he comes and we disagree it means verbal exchange of all kinds [Laugh] I talk and talk he gets angry and leave only to return very late at night - -.” (NC, age 24, Kariobangi)* *“It was worse until hey! I used to keep quiet so much because I used to feel whenever somebody talks to me just a little then I get angry and I just wish that we fight [Laughter] I mean I used to get extremely angry- -.” (JA, age 22, Kariobangi)* *“I was depressed but I had several regrets as I was just questioning myself but nowadays it is over. The regrets were 'why have I become pregnant early?” (LA, age 19, Kariobangi)* *“I am another one; whenever someone could anger me just a little I could moody, sitting in the bed with tears, and when I cook the food cannot even be eaten because of too much salt [Laughter] or raw ugali; but for now I have changed and I am good, even if you talk I act like I am not hearing or I go and sit outside and come back when he is quiet.” (JN, age 21, Kariobangi)* *“Before I joined this group, I was so depressed; I used to be an angry person, my anger was so much near, even if someone wrongs me where we live then I carry it into the house on the kids and even on the husband; but since I came here, I have changed.” (LA, age 19, Kariobangi)* *“- - at the moment I am stress-free and I just know how I can handle it; I sleep and wake up when I have forgotten those things but I normally thank God because I have never had stress.” (RJ, age 23, Kangemi)*

Adolescent participants appreciated that IPT-G helped them alleviate depressive symptoms (social isolation, anger, hopelessness and low mood). Most of them reported better sleep patterns, good appetite, increased social interactions and decreased HIV-related stigma.

*It has helped me when I am with people, I have accepted myself the way I am and then anger issues nowadays are not there, at least I can make friends; in the past, I could not make friends, but now at least I can sit with somebody and share something with her that is helpful* (SA, age 23, Kariobangi).

Isolation was one of the maladaptive behaviours associated with depressive symptoms as indicated by one of the adolescent mothers:

*I used to lock myself in the house, but currently, I can get out and make stories with neighbours, when I am called for a job I can go* (RA, age 18, Kangemi).

*I give back thanks for he has brought us from far because right now I could be in the ground [Laughter], so may God give him [YO] strength for him to continue with that spirit* (PN, age 24, Kariobangi).

We noted the devastating effects of depression as highlighted by one of the participants on how isolation, marital conflict and persistent distress used to affect her daily living. See a vignette from our participants here highlighting this point further:

*I am FW; it has helped me, I am not the same as I used to be; I used to be angry; I could get out of the house at night due to anger. Now we talk well in the house … yes [Laughter]* (FW, age 21, Kariobangi).

*I think I was depressed; I used to sit in the house and had no friends. But when I started coming to this group, I found friends here, outside I have also made friends. I used to be stressed as to why my husband doesn’t go to work and [had] frequent disagreements. But since I started coming here for advice, I realized that I am not the only one who has problems, so for me, it has gotten out; I just feel that it has helped me a lot, stress is usual, but for now, I feel stress free* (FW, age 21, Kariobangi).

Our study acknowledges that adolescents living with HIV are vulnerable to negative community perceptions. These could manifest itself with suicidal ideations:

*At the first time, I wanted to kill myself [inaudible], I have children and they could become orphans. The moment I came in this group, it has helped me until now my children are grown, and I have even started working harder because the other one is in grade two, the other is still little, but you advised me* (PN, age 24, Kariobangi).

One of the participants acknowledged that the therapy sessions improved her interpersonal relationship with her male partner and could enjoy functional changes in her life.

*We have agreed with my husband that I am going back to school…. In the past, I used to wonder how he views me* (JI, age 21, Kangemi).

On loss and grief, some of the CHWs expressed their experiences on how IPT-G helped them in processing their thoughts and emotions related to loss and grief in the past:

*It has helped me a lot, the second thing let me say I was given a husband by Kangemi people, when we were doing training, and I lost the husband [refers to CA, deceased CHV], so IPT helped me to go through the loss and grief, anyway it was not a real husband [Laughter]. But he was a very good friend of mine. Anyway, when I was undergoing through the sessions, I lost most of [my] very important people, but IPT helped me. You know when we were talking to these girls, and they are also expressing,* ‘*I lost my kid, I lost my daddy,*’ *and I was also like it was like me losing the people whom I care[d] about, so we were going through these together. It was a process for all of us, so it healed me, and it healed them; that was very good* (EO, age 24, CHV, Kariobangi).

The CHA was mourning the loss of her child during the sessions and had to say this in appreciation of the intervention:

… *and when I came here, I was so much stressed, and it could have resulted in depression because there is something, I lost my child on delivery, then I lost both parents at the same time. So, when we started talking, I felt that I had put the load down, you get it? So when we continued and reached the middle, and I realized that I am good* (NG, age 43, CHV, Kangemi).

We were able to form a strong collaborative team by creating a WhatsApp group and took issues of the CHWs and the PPAs with a great deal of importance by demonstrating practical empathy, thus improving commitment for all of us in the study process.

*When I lost my child on delivery, I also want to thank YO, I don’t know what to tell him, but there is a time I looked and realized that he is a very different person. We hadn’t known each other—we had known each other for only three days, but when I had a problem he came with MK to my house and consoled me, and I felt that it wasn’t even about friendship, they are just part of like my family, and he helped me* (LN, age 34, CHA, Kangemi).

The loss of one of the CHVs affected most of our participants emotionally and it could be because he was youthful and easily identified with him during subsequent sessions. Besides, we realized that most of our CHWs had experienced the loss of a loved one and were also struggling with healing, which our sessions also supported them as they narrated to us. The CHWs were affected by the loss of a colleague (CA), and in addition to other previous bereavements that worsened their state [42], and IPT-G helped them cope:

*CA also left me, he was my friend, and it drew me so much down, but now I am fine, isn’t it?* … *During CA’s time of demise, it was like everybody in Kangemi felt like was carrying something [Silence]* (LN, age 34, CHA, Kangemi).

The lead researcher, clinical supervisor and research assistant organized a loss and grief therapy session for the group (adolescents and CHWs) using one of the IPT problem areas. We also visited the family and arranged for tree planting within the facility whereby family members and clinical staff were invited for the function as a means of bringing closure for all who knew the deceased CHV. We notified the ethics office of the incident which was documented in our protocol.

At both study sites, there was a shortage of trained mental health personnel. Kangemi health centre has only one nurse in training under the sponsorship of a non-governmental organization. At the Kariobangi health centre, there are only two health care workers with post-diploma training in psychiatry. There was no mental health designated space, and counsellors under HIV testing services (HTS) were operating from mobile tents.

Nevertheless, several CHWs acknowledged the lack of a designated space for mental health services as one of the limitations towards adopting IPT-G delivery to routine PMTCT services:

*I think for us [in] Kariobangi, we had a big challenge when it came to the venue of the meeting because sometimes you find we are here sometimes we are displaced at the tent, maybe the other tent is very dirty, sometimes we are in this other tent we come here displaced, so it was a very big challenge [cross talk]* …. (EO, age 24, CHV, Kariobangi).

The space to administer mental health services was missing within the health centres as shown by how we had to improvise a room for the sessions:

*We were okay; we had been given the maternity, a place somewhere…. You see, if we close the middle of the room too and pull the curtains, it was so good; it didn’t have an issue* (LN, age 34, CHA, Kangemi).

Another CHW seemed helpless because of the situation and was contented to the state of the available space despite all the challenges associated with it:

*But let’s just say it was okay; even if it is bad, it is our place, so we cannot say that it is bad [laughter], but it was fine* (JA, age 24, CHV, Kangemi).

The lack of a specified venue for mental health services caused uncertainty for effective service delivery for the CHWs:

*The space was okay; it was somewhere where we don’t have issues with noise, but we don’t know next* (SN, age 65, CHV, Kariobangi).

### Opportunity

**Fig. 2:**
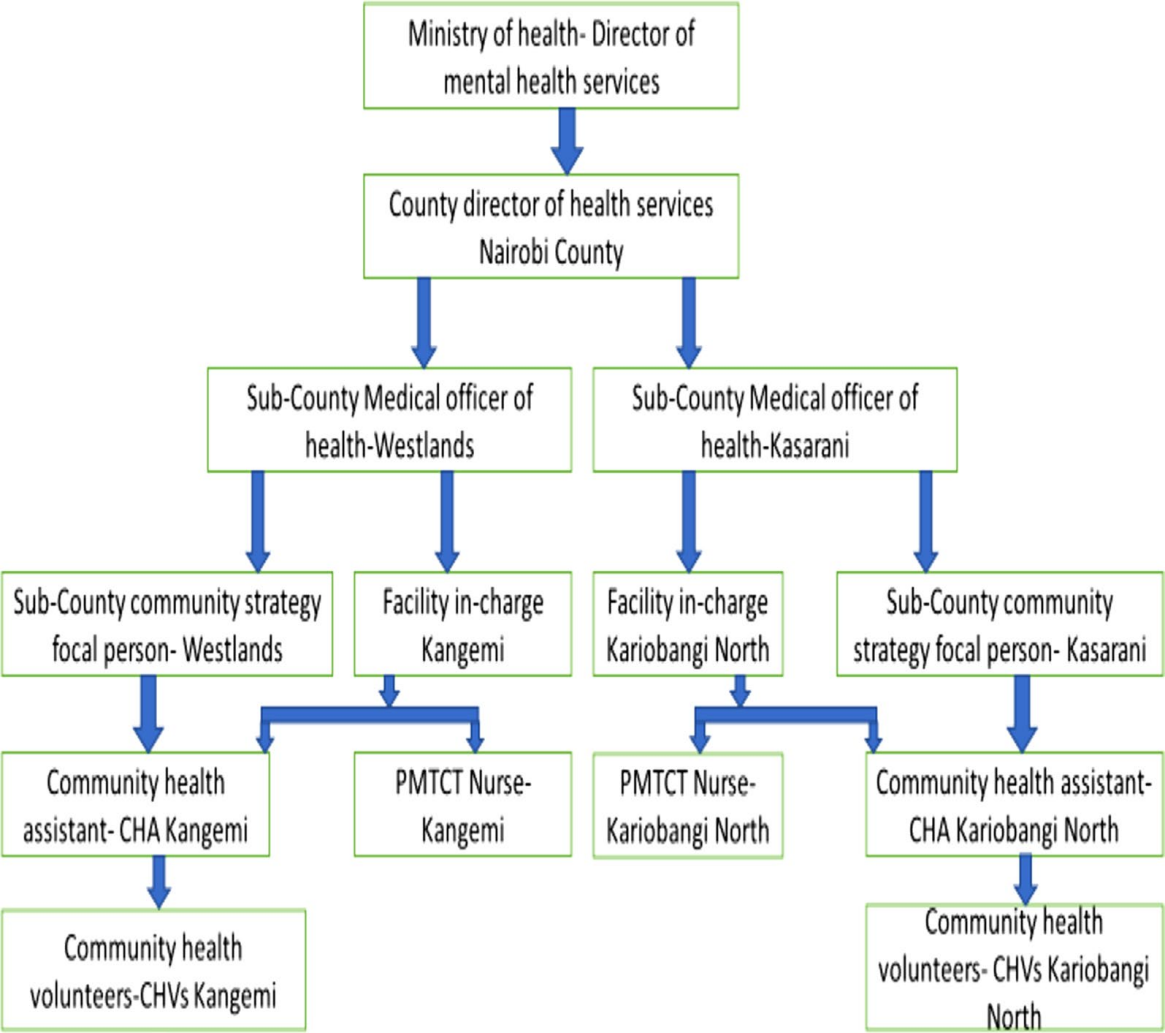
Collaborative structure for IPT implementation

Capacity building through a collaborative care approach was used, which involved engaging the Director of Mental Health Services and cascading the partnership along with the health workers downwards to the level of the CHWs. The entire health workers were very supportive of our intent by issuing us with clearance and linking us to all the relevant clinical staff (see Fig. 2).

Screening questionnaires were administered to the CHWs before training to assess their knowledge of mental health concepts such as stress and depression. They all demonstrated a fair understanding of the difference between these two concepts. The CHWs appreciated the continuous supportive supervision and they felt that all their concerns arising from the weekly sessions were being addressed promptly and adequately, thus enhancing their competency to deliver IPT-G:

*Follow-up of that client, you have done IPT for this client and you have done termination session; you have referred that client from facility to the community, who will do that follow-up. The community health workers. They are the linkage between the facility and the community and community to the facility (LN,age 34,CHA,Kangemi).*

The nursing officer in-charge in one of the health centers was able to appreciate how IPT-G was being embraced by the mentor-mother within the facility:

*She is very committed and goes to other areas and departments to help the clients and train the clients. She often goes out of her way to serve the patients which shows interest for many* (*SK, age 46, Kangemi).*

Some of the resources required for roll-out of IPT-G according to a PMTCT nurse in of the study sites were:

*Space for to conduct IPT, time for both PPAs and CHWs, need for snacks and need also to motivate the CHVs to organize and conduct(JM,age 38,PMTCT Nurse,Kangemi).*

The medical laboratory technologist was able to appreciate IPT-G for the PPAs citing younger age being a risk factor to higher viral load:

*Pregnancy at younger age and misconceptions associated with ART will affect their adherence hence make their viral load to go higher (AM, age 32, Lab Tech,Kangemi).*

The CHWs felt more aware of the needs of the PPAs after the group sessions:

*In fact we always talk about that group (IPT participants) wherever I go and maybe I meet a partner. I am still looking for a partner to support them. I always talk about it because we felt that we changed them and we wouldn't like them to get lost on the way, we would love them to come back and help the others (LN,age 34,CHA,Kangemi).*

Furthermore, it was very encouraging to hear from the CHWs that our participants after intervention became role models in the community by imparting skills in management of their day-to-day issues of life (see Table 5).

**Table 5:** CHWs understanding of IPT-G intervention

IPT themes	Quotations
*Addresses loss and grief for CHWs*	*“Me too it has helped me. Now that I can talk to them. I used to see a pregnant woman and I take another route that I don't want to meet. Where there were children, I could not go, but now I even carry your kids so it has helped me that's why you can see how strong I am.”(LN,age 34,CHA,Kangemi)*
*Empowered PPAs to live a better life*	*“It has been a nice thing, every time I see you. I normally get impressed and just smile because at least there is progress in our lives, we are not the same as the way we used to be okay?” (EO, age 24,CHV, Kariobangi)* *“I think that will help a lot because you see for the young girls you might find that when they do HIV test the husband is negative and the lady is positive so you see the girl gets stressed because she is harassed by the husband "where did you get the virus?" And whatever, it will help a lot [Silence].” (JA,age 24,CHV,Kangemi)*
*Building capacity and acknowledging the team*	*“Follow-up of that client, you have done IPT for this client and you have done termination session; you have referred that client from facility to the community, who will do that follow-up? The community health workers. They are the linkage between the facility and the community and community to the facility.” (LN,age 34,CHA,Kangemi)*
*Third IPT session is when disclosure happens*	*“- -yes, they exist; when we started, we had a challenge because one we had not been trained, we had a challenge but we reached where we reached. I can say that even those girls when we start with them there are usually problems but the good thing is that mid-way they pick and can listen to you and sessions go well.” (JA,age 24,CHV,Kangemi)*
*IPT Impact in the community*	*“In fact we always talk about that group (IPT participants) wherever I go and maybe I meet a partner. I am still looking for a partner to support them. I always talk about it because we felt that we changed them and we wouldn't like them to get lost on the way, we would love them to come back and help the others.” (LN,age 34,Kangemi)* *“I think there is a time when we began IPT, those girls most of them were locking themselves in the house.…. but now after the sessions in Kariobangi, we realized that they went back to work. Most of them have gone back to work, some other have started businesses, MA got a job and also has a business, EL has a business. So, people have gone back to work, there is SA has gone back to work; people are going back to work, people have started businesses but for one or two we are still following.” (EO, age 24, Kariobangi)*
*Group strengthens cohesion and empowers*	*“She (PPA) realizes that she is not the only one and that they are several, don't you even see that the first thing they were accepting so much whenever she hears that this one is also like me, even this one and the other one so she feels that 'we are many.” (NG,age,CHV.43,Kangemi)* *“It used to help them to unwind because maybe somebody has an issue and feels like 'maybe it is only me who has this issue' so when she finds somebody in the group who says almost like similar to hers then she also opens her heart and speaks.” (EO, age 24,CHV. Kariobangi)*

## Discussion

In this study, we were interested in understanding the behaviour of the CHWs towards the delivery of IPT-G within primary health care settings. We utilized a theoretical framework of behaviour change: Capacity, Opportunity and Motivation to understand our findings. A similar study had previously used the COM-B framework to assess the barriers and facilitators to the integration of mental health services into primary health care [43].

Our study highlighted the possible benefits accrued from IPT-G for both the CHWs and the PPAs including: positive impacts on individuals and the community, improved interpersonal relationship, reduction in depressive symptoms, loss and grief therapy, barriers to IPT-G delivery, capacity building and skills development for the staff within the PMTCT clinic at the primary health care. The CHWs found the intervention useful in addition to their existing knowledge and skills with enough competence to deliver IPT-G. Our research team noted that IPT-G can be disseminated to other similar settings by utilizing CHWs [44]. We agree that trained and supervised CHWs can equally deliver this intervention [45, 46]. In cognizance of universal health care, we too lend our voice towards the need to consider the CHWs to be involved in mental health care delivery to cover for the shortage of trained mental health specialists [36].

The IPT-G is a brief psychotherapy that is good for depressed adolescents since it addresses developmental tasks, clinical aspects of depression and treatment barriers [47]. Supportive supervision for the CHWs is necessary for effective task shifting. It describes the process of strengthening relationships within the health care system with a focus on problem-solving, allocation of necessary resources, promoting teamwork and open communication with a view to improving service delivery [48].

Adolescent depression has a devastating impact on school functioning, social behaviour, and family relationships which IPT-G has demonstrated improvement in overall functioning [49]. Our adolescent participants narrated the benefits of this intervention considering they were now able to effectively communicate, interact with others, manage their anger and even resume work for an income. It is worth noting that, a previous study which assessed interpersonal relationships between the youth and their families or caregivers, it was found that poor relationships were strongly related to depression symptoms [50, 51]. The adaptation of IPT-G for depressed adolescents yielded good results with the advantage of them utilizing the group as a place for experimenting with new ways of communication and interacting with others [33]. A study in Uganda where IPT-G was delivered by the CHWs to caregivers of children affected by nodding syndrome in low-resource government facilities affirms the same benefits. Significant impact was observed for psychological distress, stigma, and social support [52].

Task shifting for IPT-G has proved to be effective when delivered by supervised CHWs as noted in South Africa where primary health care users with moderate to severe depression showed a significant reduction in depressive symptoms. The qualitative process suggested the reduction of depressive symptoms could have been due to improved social support, individual coping skills, and improved personal agency [53]. A grand study within India, Ethiopia, Nepal, South Africa, and Uganda to assess the acceptability and feasibility when non-specialist workers to delivers mental interventions reported positive findings. They recommended that for task shifting to succeed in LMICs, there is a need for the allocation of more human resources in primary health care clinics, structured supportive supervision for non-specialist health workers, adequate training and compensation for health workers involved in task shifting [54].

The adaptation of IPT-G for people living with HIV/AIDS in Northwest Ethiopia also found it acceptable when delivered by trained peer counsellors. The qualitative study identified factors associated with depression as psychosocial problems, spiritual factors and biological factors, and highlighted its impact at the individual and family level [55]. The CHWs are an integral part of primary health care within LMICs as seen in addressing the COVID-19 pandemic and this could also apply to other illnesses. They played a significant role in health education, contact tracing and mobilization during vaccination. In the end, this was a cost-saving and effective way of reaching a majority of the population [56]. Culturally and developmentally, the adaptation of IPT-G for depressed adolescents has previously been conducted in Nepal [57].

Our study population was noted to have several challenges (poor sleep patterns, poor appetite, impaired social interactions, and HIV-related stigma),which seemed to support previous studies where they found out that adolescents living with HIV are more vulnerable to mental health disorders [58, 59]. There is no doubt that our intervention addressing depression will improve adherence to ART among our study participants, which is key to a good quality of life [28].

One qualitative study suggested that PPAs living with their partners or caregivers will benefit from social support as they navigate early motherhood challenges [60].Our study acknowledges that adolescents living with HIV are vulnerable to negative community perceptions which agrees with a similar study in Uganda [61]. Spouses supporting PPAs with financial and social provision to return to school are likely to minimize future unplanned or unwanted pregnancies and enable them to acquire better lives for themselves and even for their children [62].

HIV affects adolescents and its unpleasant outcomes are indirectly transferred to the child [63], as shown by one of the participants who felt that she could have died of a stressful life leaving her child, but the sessions helped her. Early sexual intimacy with the opposite gender predisposes girls to unplanned and unwanted pregnancies with the possibility of dropping out of school and even lacking skills to gain employment [64, 65]. Research has shown that early unintended pregnancy is a risk factor for later HIV infections among adolescents [13]. HIV-related stigma has been associated with difficulties in adhering to ART [26, 27].

Continuous supportive supervision was appreciated by the CHWs and they felt that all their concerns arising from the weekly sessions were addressed promptly and adequately. During our group sessions, supportive supervision emphasized on joint problem-solving, mentoring and two-way communication between the researchers and the CHWs [66, 67].

It was exciting to hear from the CHWs that our participants after intervention became role models in the community by imparting the skills of managing their day-to-day life issues. For both CHWs and adolescents, the learning cycle was achieved by both actual experiences and empowering them to practice new pleasant skills during the sessions [68, 69].

Further studies is recommended on the use of digital technological approach to deliver this intervention using similar methodology among adolescent mother living with HIV [30].This intervention is targeting the right population considering that most of the perinatal adolescents face several difficulties such as social stigma, lack of emotional support, poor health care access and stresses around new life adjustments [70]. Our IPT-G intervention helped adolescents by improving their interpersonal relationships, communication processes and overall mental health.

### Strengths

We were able to qualitatively establish the acceptability and feasibility of IPT-G delivered to PPAs living with HIV by the CHWs in primary health care settings. The two study sites make the findings more reliable for a representative outcome for low-resource urban settings.

### Limitations

Despite all the exciting positive impact on the participants’ lives from this intervention as seen from the qualitative findings, the sample size was small hence not powered.

## Conclusion

The shortage of trained mental health workers has led to the inaccessibility of mental health services both in urban and rural settings. To enhance the availability of mental health services to the broader population, our study sought to assess the acceptability and feasibility of IPT-G being delivered to PPAs living with HIV by the CHWs within routine clinical settings. Our findings affirm that this intervention has a promise of being delivered by the CHWs for this specific population and the need for supportive supervision to enhance fidelity. We recommend a follow-up study with a larger representative sample size from a more diverse community within urban and rural settings with a view to scaling up IPT-G at the primary health care level in all counties.

## Supplementary Information

**Additional file 1. Table 6**. Consolidated Criteria for Reporting Qualitative Studies hence not part of the text but only serves as a checklist during manuscript writing.

## Data Availability

The data collected is in the form of qualitative format and bulky hence are available from the corresponding author on request.

## Abbreviations

CHA: Community health assistant
CHV: Community health volunteer
EPDS: Edinburgh Postnatal Depression Scale
IPT-G: Group Interpersonal Psychotherapy
HIV: Human immunodeficiency virus
LMICs: Low-and middle-income countries
PMTCT: Prevention of mother-to-child transmission
PPA: Postpartum adolescents
PPD: Postpartum depression

## References

[1] World Health Organization (WHO). Task Shifting: global recommendations and guidelines Geneva, Switzerland; 2007.

[2] World Health Organization (WHO). Treat, train, retain: the AIDS and health workforce plan: report on the consultation on AIDS and human resources for health, WHO, Geneva, 11–12 May, 2006. IAPAC Mon. 2006;12(5):144–6. 17249139

[3] Dawson AJ, Buchan J, Duffield C, Homer CSE, Wijewardena K (2014). Task shifting and sharing in maternal and reproductive health in low-income countries: a narrative synthesis of current evidence. Health Policy Plan.

[4] Mundeva H, Snyder J, Ngilangwa DP, Kaida A (2018). Ethics of task shifting in the health workforce: exploring the role of community health workers in HIV service delivery in low- and middle-income countries. BMC Med Ethics.

[5] Mwai GW, Mburu G, Torpey K, Frost P, Ford N, Seeley J (2013). Role and outcomes of community health workers in HIV care in sub-Saharan Africa: a systematic review. J Int AIDS Soc..

[6] Angwenyi V, Kamuya D, Mwachiro D, Marsh V, Njuguna P, Molyneux S (2013). Working with Community Health Workers as “volunteers” in a vaccine trial: practical and ethical experiences and implications. Dev World Bioeth.

[7] UNAIDS and Stop AIDS Alliance. Communities deliver. 2015;23–80.

[8] UNAIDS. Global AIDS update 2018 miles to go: Closing gaps breaking barriers righting injustices. UNAIDS [Internet]. 2018. http://www.unaids.org/sites/default/files/media_as. Available from: http://www.unaids.org/sites/default/files/media_asset/miles-to-go_en.pdf.

[9] NACC. www.Nacc.or.Ke. Progress, Response. 2016.

[10] NASCOP - Kenya 2015. Kenya AIDS estimates, 2015. 2016. www.nascop.or.ke.

[11] Kassa GM, Arowojolu AO, Odukogbe AA, Yalew AW (2018). Prevalence and determinants of adolescent pregnancy in Africa: a systematic review and Meta-analysis. Reprod Health.

[12] UNAIDS. In sub-Saharan Africa, three in five new HIV infections among 15–19-year-olds are among girls. 2019;2019. https://www.unaids.org/sites/default/files/women_girls_hiv_sub_saharan_africa_en.pdf.

[13] Christofides NJ, Jewkes RK, Dunkle KL, Nduna M, Shai NJ, Sterk C (2014). Early adolescent pregnancy increases risk of incident HIV infection in the Eastern Cape, South Africa: a longitudinal study. J Int AIDS Soc..

[14] de Oliveira T, Kharsany ABM, Gräf T, Cawood C, Khanyile D, Grobler A (2017). Transmission networks and risk of HIV infection in KwaZulu-Natal, South Africa: a community-wide phylogenetic study. Lancet HIV.

[15] Kim MH, Mazenga AC, Devandra A, Ahmed S, Kazembe PN, Yu X (2014). Prevalence of depression and validation of the Beck Depression Inventory-II and the Children’s Depression Inventory-Short amongst HIV-positive adolescents in Malawi. J Int AIDS Soc.

[16] Kamau JW, Kuria W, Mathai M, Atwoli L, Kangethe R (2012). Psychiatric morbidity among HIV-infected children and adolescents in a resource-poor Kenyan urban community. AIDS Care.

[17] Nunes AP, Phipps MG (2013). Postpartum depression in adolescent and adult mothers: comparing prenatal risk factors and predictive models. Matern Child Health J.

[18] Torres R, Goyal D, Burke-Aaronson AC, Gay CL, Lee KA (2017). Patterns of symptoms of perinatal depression and stress in late adolescent and young adult mothers. J Obstet Gynecol Neonatal Nurs.

[19] Dinwiddie KJ, Schillerstrom TL, Schillerstrom JE (2018). Postpartum depression in adolescent mothers. J Psychosom Obstet Gynecol.

[20] Sangsawang B, Wacharasin C, Sangsawang N (2019). Interventions for the prevention of postpartum depression in adolescent mothers: a systematic review. Arch Womens Ment Health.

[21] Sherr L, Clucas C, Harding R, Sibley E, Catalan J (2011). HIV and depression—a systematic review of interventions. Psychol Health Med.

[22] Cruess DG, Evans DL, Repetto MJ, Gettes D, Douglas SD, Petitto JM (2003). Prevalence, diagnosis, and pharmacological treatment of mood disorders in HIV disease. Biol Psychiatry.

[23] Martinez J, Chakraborty R, Aldrovandi GM, Chadwick EG, Cooper ER, Kourtis A (2014). Psychosocial support for youth living with HIV. Pediatrics.

[24] Schunter BT, Cheng W-S, Kendall M, Marais H (2014). Lessons learned from a review of interventions for adolescent and young key populations in Asia Pacific and opportunities for programming. JAIDS J Acquir Immune Defic Syndr.

[25] Januraga PP, Mooney-Somers J, Ward PR (2014). Newcomers in a hazardous environment: a qualitative inquiry into sex worker vulnerability to HIV in Bali, Indonesia. BMC Public Health.

[26] Ammon N, Mason S, Corkery JM (2018). Factors impacting antiretroviral therapy adherence among human immunodeficiency virus–positive adolescents in sub-Saharan Africa: a systematic review. Public Health.

[27] Croome N, Ahluwalia M, Hughes LD, Abas M (2017). Patient-reported barriers and facilitators to antiretroviral adherence in sub-Saharan Africa. AIDS.

[28] Sin NL, DiMatteo MR (2014). Depression treatment enhances adherence to antiretroviral therapy: a meta-analysis. Ann Behav Med.

[29] Wagner GJ, Ghosh B, Barbara D, Sebastian M (2020). Changes in ART adherence relate to changes in depression as well: evidence for the bi-directional longitudinal relationship between depression and ART adherence from a prospective study of HIV clients in Uganda. AIDS Behav.

[30] Dulli L, Ridgeway K, Packer C, Plourde KF, Mumuni T, Idaboh T (2018). An online support group intervention for adolescents living with HIV in Nigeria: a pre-post test study. JMIR Public Heal Surveill.

[31] Okawa S, Mwanza Kabaghe S, Mwiya M, Kikuchi K, Jimba M, Kankasa C (2018). Psychological well-being and adherence to antiretroviral therapy among adolescents living with HIV in Zambia. AIDS Care Psychol Socio Med Asp AIDS/HIV.

[32] Bhana A, Mellins CA, Small L, Nestadt DF, Leu CS, Petersen I (2016). Resilience in perinatal HIV+ adolescents in South Africa. AIDS Care Psychol Socio Med Asp AIDS/HIV.

[33] Mufson L, Gallagher T, Dorta KP, Young JF (2004). A group adaptation of interpersonal psychotherapy for depressed adolescents. Am J Psychother.

[34] WHO. WHO | WHO called to return to the Declaration of Alma-Ata. WHO. 2017.

[35] WHO-Unicef. ALMA-ATA Primary Health Care. International conference on primary health care, vol. 63. 1978.

[36] Ministry of Health. Kenya Community Health Policy 2020–2030. 2020.

[37] Michie S, van Stralen MM, West R (2011). The behaviour change wheel: A new method for characterising and designing behaviour change interventions. Implement Sci.

[38] WHO, Columbia U. Group Interpersonal Therapy (Ipt) for depression, vol 100. World Heal Organ [Internet]. 2016. www.who.int/about/licensing/copyright_form/en/index.html.

[39] Cox JL, Holden JM, Sagovsky R (1987). Detection of postnatal depression. Br J Psychiatry.

[40] McCain GC (1988). Content analysis: a method for studying clinical nursing problems. Appl Nurs Res.

[41] Sekhon M, Cartwright M, Francis JJ (2017). Acceptability of healthcare interventions: an overview of reviews and development of a theoretical framework. BMC Health Serv Res.

[42] Shear MK (2012). Grief and mourning gone awry: pathway and course of complicated grief. Dialogues Clin Neurosci.

[43] Wakida EK, Obua C, Rukundo GZ, Maling S, Talib ZM, Okello ES (2018). Barriers and facilitators to the integration of mental health services into primary healthcare: a qualitative study among Ugandan primary care providers using the COM-B framework. BMC Health Serv Res.

[44] World Health Organization (WHO) (2008). Task shifting. Global Recomendations and guidelines.

[45] Murray KR, Dulli LS, Ridgeway K, Dal Santo L, De Mora DD, Olsen P (2017). Improving retention in HIV care among adolescents and adults in low- and middle-income countries: a systematic review of the literature. PLoS One..

[46] Kredo T, Adeniyi FB, Bateganya M, Pienaar ED. Task shifting from doctors to non-doctors for initiation and maintenance of antiretroviral therapy. In: Cochrane database of systematic reviews, vol 2014. Hoboken: Wiley; 2014. 10.1002/14651858.CD007331.pub3 PMC11214583 24980859

[47] Moreau D, Mufson L (1997). Interpersonal psychotherapy for depressed adolescents. Child Adolesc Psychiatr Clin N Am.

[48] Kemp CG, Petersen I, Bhana A, Rao D (2019). Supervision of task-shared mental health care in low-resource settings: a commentary on programmatic experience. Glob Health Sci Pract.

[49] Mufson L, Dorta KP, Wickramaratne P, Nomura Y, Olfson M, Weissman MM (2004). A randomized effectiveness trial of interpersonal psychotherapy for depressed adolescents. Arch Gen Psychiatry.

[50] Miller L, Hlastala SA, Mufson L, Leibenluft E, Riddle M (2016). Interpersonal psychotherapy for adolescents with mood and behavior dysregulation: Evidence-based case study. Evid Based Pract Child Adolesc Ment Health.

[51] Spence SH, O’Shea G, Donovan CL (2016). Improvements in interpersonal functioning following interpersonal psychotherapy (IPT) with adolescents and their association with change in depression. Behav Cogn Psychother.

[52] Mutamba BB, Kane JC, De Jong JTVM, Okello J, Musisi S, Kohrt BA (2018). Psychological treatments delivered by community health workers in low-resource government health systems: effectiveness of group interpersonal psychotherapy for caregivers of children affected by nodding syndrome in Uganda. Psychol Med..

[53] Petersen I, Bhana A, Baillie K, MhaPP Research Programme Consortium. The feasibility of adapted group-based interpersonal therapy (IPT) for the treatment of depression by community health workers within the context of task shifting in South Africa. Community Ment Health J. 2012;48(3):336–41. 10.1007/s10597-011-9429-2 21687982

[54] Mendenhall E, De Silva MJ, Hanlon C, Petersen I, Shidhaye R, Jordans M (2014). Acceptability and feasibility of using non-specialist health workers to deliver mental health care: stakeholder perceptions from the PRIME district sites in Ethiopia, India, Nepal, South Africa, and Uganda. Soc Sci Med.

[55] Asrat B, Lund C, Ambaw F (2020). Schneider M Adaptation of the WHO group interpersonal therapy for people living with HIV/AIDS in Northwest Ethiopia: a qualitative study. PLoS ONE..

[56] Mistry SK, Harris-Roxas B, Yadav UN, Shabnam S, Rawal LB, Harris MF. Community health workers can provide psychosocial support to the people during COVID-19 and beyond in low- and middle-income countries. Front Public Heal. 2021;9. 10.3389/fpubh.2021.666753 PMC8258154 34239854

[57] Rose-Clarke K, Pradhan I, Shrestha P, Prakash BK, Magar J, Luitel NP (2020). Culturally and developmentally adapting group interpersonal therapy for adolescents with depression in rural Nepal. BMC Psychol..

[58] Nanni MG, Caruso R, Mitchell AJ, Meggiolaro E, Grassi L (2015). Depression in HIV infected patients: a review. Curr Psychiatry Rep.

[59] Mellins CA, Malee KM (2013). Understanding the mental health of youth living with perinatal HIV infection: lessons learned and current challenges. J Int AIDS Soc.

[60] UNESCO. Early and unintended pregnancy & the education sector (ED/IPS/HAE/2517/01REV). Paris. 2017. http://unesdoc.unesco.org/images/0025/002515/251509E.pdf.

[61] Ashaba S, Cooper-Vince CE, Vořechovská D, Rukundo GZ, Maling S, Akena D (2019). Community beliefs, HIV stigma, and depression among adolescents living with HIV in rural Uganda. Afr J AIDS Res.

[62] Human Rights Watch. Africa: pregnant girls, young mothers barred from school. Human Rights Watch [Internet]. 2018 [cited 2020 May 15]. https://www.hrw.org/news/2018/06/14/africa-pregnant-girls-young-mothers-barred-school.

[63] González R, Rupérez M, Sevene E, Vala A, Maculuve S, Bulo H (2017). Effects of HIV infection on maternal and neonatal health in southern Mozambique: a prospective cohort study after a decade of antiretroviral drugs roll out. PLoS ONE..

[64] Erken A, Schensul BD. US of the WP. 2019. https://www.unfpa.org/sites/default/files/pub-pdf/UNFPA_PUB_2019_EN_State_of_World_Population.pdf.

[65] Schensul BD. UNFPA State of the World Population. 2019. https://www.unfpa.org/sites/default/files/pub-pdf/UNFPA_PUB_2019_EN_State_of_World_Population.pdf.

[66] Lehmann U, Sanders D. Community health workers: what do we know about them? The state of evidence on programmes, activities, costs and impact on health outcomes of using community health workers. 2007.

[67] Lewin SA, Dick J, Pond P, Zwarenstein M, Aja GN, van Wyk BE, et al. Lay health workers in primary and community health care. In: Lewin SA, editor. Cochrane Database of SystematicReviews. John Wiley & Sons, Ltd; 2005. 10.1002/14651858.CD004015.pub2 15674924

[68] Huber GP (1991). Organizational learning: the contributing processes and the literatures. Organ Sci.

[69] Newell S (2005). Knowledge transfer and learning: problems of knowledge transfer associated with trying to short-circuit the learning cycle. JISTEM J Inf Syst Technol Manag.

[70] Kumar M, Huang K-Y, Othieno C, Wamalwa D, Madeghe B, Osok J (2018). Adolescent pregnancy and challenges in kenyan context: perspectives from multiple community stakeholders. Glob Soc Welf.

