# Supplementary material for: Acceptability and impact of group interpersonal therapy (IPT-G) on Kenyan adolescent mothers living with human immunodeficiency virus (HIV): a qualitative analysis
Source: BMC Womens Health. 2022 Jun 18;22:240. doi: 10.1186/s12905-022-01807-w (PMC9206094; doi:10.1186/s12905-022-01807-w)
Supplement: Supplementary file 1 — Additional file 1. Table 6. Consolidated Criteria for Reporting Qualitative Studies hence not part of the text but only serves as a checklist during manuscript writing. [file 12905_2022_1807_MOESM1_ESM.docx]

**Table 6: Consolidated criteria for reporting qualitative studies (COREQ): 32-item checklist**

| *No.* | *Item* | *Reference section, page and paragraph* |
| --- | --- | --- |
|  | **Domain 1: Research team and reflexivity** |  |
|  | **Personal Characteristics** |  |
|  | Interviewer/facilitator | Obadia Yator (Please see page 1 authors section) |
|  | Credentials | MSc in Clinical Psychology, Ph.D. candidate in Clinical Psychology, Department of Psychiatry, School of Medicine, College of Health Sciences, University of Nairobi (Please see page 1 authors section) |
|  | Occupation | Clinical psychologist within a government hospital in Kenya (Please see page 1 authors affiliations). |
|  | Gender | Male (Please page 1 authors section-Obadia is a name for male gender) |
|  | Experience and training | Practicing psychologist with over ten (10) years’ experience (Please see page 1 authors section- PhD holder in Clinical Psychology). |
|  | **Relationship with participants** |  |
|  | Relationship established | Rapport was established during recruitment where individualized engagement was done (Please see page 8, paragraph 4, methods section on ethical approval and consent from the participants-. Both study sites had same numbers of CHWs with all participants signing a written informed consent |
|  | Participant knowledge of the interviewer | Purpose of the study was shared with the participants and requested them to sign written informed consent. (Please see page 8, paragraph 4, methods section on ethical approval and consent from the participants-Purpose and voluntary participation was discussed with participants before incitation the intervention). |
|  | Interviewer characteristics | All participants were reassured of shared confidentiality and reassured them of equal opportunity to participate during the sessions. (Please see page 9,paragraph 3 up to page 10, paragraph 1, methods section on ethical approval and consent from the participants-Purpose and voluntary participation was discussed with participants before initiation of the intervention). |
|  | **Domain 2: study design** |  |
|  | **Theoretical framework** |  |
|  | Methodological orientation and Theory | Our study adopted theoretical framework of behavior change; Capacity, Opportunity and Motivation to help understand behavior changes due to IPT-G intervention being delivered by CHWs to Kenyan adolescent mothers living with HIV(37). Acceptability and impact of IPT-G when delivered by lay workers were identified by using this framework (see figure 2). In this framework, capability (building skills on IPT-G), motivation (competencies in IPT-G) and opportunity (provision of continuous supportive supervision) will enact a behavior (Improved outcomes for adolescents, and improved health system and community strengthening).  We involved all the relevant stakeholders in the ministry of health as shown on Figure 1: Collaborative structure for IPT implementation.(Please see page 7,last paragraph up to page 8 paragraph 1) |
|  | **Participant selection** |  |
|  | Sampling | For our qualitative findings, purposeful sampling was used after the intervention to recruit PPAs (n=19) and CHWs (n=7) for FGDs to allow them to share their experiences and perception about IPT-G. The FGDs were conducted among CHWs (2 pre-trial within each in respective study sites and 1 post-trial with combination of all CHWs).Three audio-recordings from the eight IPT-G sessions was randomly selected from each study site to inform on their experiences and observations. In-depth interviews were conducted among the medical staff within the two sites: nursing officer-in-charge (n=2), mentor-mother (n=2), laboratory technologist (n=2), prevention of mother -to- child transmission (PMTCT)-Nurse (n = 2) and CHAs (n=2). CHWs delivered IPT-G to the two groups weekly for eight sessions as per the WHO protocol (37). (Please see page 10, paragraph 1, methods section) |
|  | Method of approach | Face-to-face engagement was during the pre-group phase where individual clinical interview was conducted. (Please see page 10, paragraph 2, methods section) |
|  | Sample size | Eight (8) CHWS delivered IPT-G among Twenty-four (24) participants/young mothers from the two primary health care facilities. (Please see page 8, paragraph 3, methods section). |
|  | Non-participation | All eight (8) CHWs who had been recruited for the study delivered IPT-G. Screened 46 Adolescent mothers living with HIV three weeks prior to the study and attending PMTCT,32 had depressive symptoms and met the criteria (EPDS>10), 25 reported back for first session of IPT-G, and one opted to drop her participation due to change of residence to distant vicinity thus remaining with 24 participants for the study.(Please see page 9, paragraph 3, methods section) |
|  | **Setting** |  |
|  | Setting of data collection | Data was collected at the prevention of Mother -to-Child Transmission clinic (PMTCT)at the primary health care setting in an urban population.(Please see page 8, paragraph 2, methods section) |
|  | Presence of non-participants | The intervention was delivered by trained Lay heath care workers (Community heath assistants and community heath volunteers). Also, during the study period, I engaged all key stake holders in the ministry heath handling on community heath including the facility Nursing officer in-charge, laboratory technologist and the lay heath care workers mentoring the adolescent’s mothers at the PMTCT. (Please see figure 1 on the structure of IPT-G intervention) |
|  | Description of sample | The delivery of IPT-G by trained lay heath care works was conducted between August 2018 and July 2019.The study site was in an urban population with most participants having low socio-economic status.(Please see page 8, paragraph 3, methods section) |
|  | **Data collection** |  |
|  | Interview guide | The questions for the in-depth interviews and FGDs were semi-structured and administered to the two study sites in a similar manner. (Please see page 10, paragraph, methods section) |
|  | Repeat interviews | FGDs among the lay health care providers was conducted before the IPT-G intervention and another one was done post-intervention. |
|  | Audio/visual recording | Audio-recording was used during in-depth interviews and FGDs. (Please see page 10, paragraph 3, and methods section) |
|  | Field notes | Field notes was written continuously at every visit and including during IPT-G sessions. (Please see page 10, paragraph 3, and methods section) |
|  | Duration | In-depth interviews on average were 30 minutes and FGDs was about 90 minutes. (Please see page 10, paragraph 1, methods section) |
|  | Data saturation | Separate FGDs was conducted among PPAs and CHWs, and In-depth interviews among key staff at the PMTCT. (Please see page 10, paragraph 1, methods section) |
|  | Transcripts returned | During the in-depth interviews and FGDs, findings arising from delivery of IPT-G was explored among the nursing officer in-charge, laboratory by technologist and lay health care providers (CHVs). The transcripts were returned to the participants upon analysis. (Please see page 10, paragraph 1, methods section) |
|  | **Domain 3: analysis and findings** |  |
|  | **Data analysis** |  |
|  | Number of data coders | Three (3) coders participated in the process whereby the lead researcher, clinical supervisor and research assistant participated in coding and thereafter the three mentors of this study looked at the overall themes generated from the study(Please see page 11,paragraph 1) |
|  | Description of coding tree | The authors picked on the observations and experiences during the study period and discussed on some of the quotations obtained during IPT-G. (Please see page 11, paragraph 1, methods section). |
|  | Derivation of themes | Our study adopted theoretical framework of behavior change. In this framework, capability (building skills on IPT-G), motivation (competencies in IPT-G) and opportunity (provision of continuous supportive supervision) will enact a behavior (Improved outcomes for adolescents, and improved health system and community strengthening).  The observations and perceptions from CHWs and PPAs during the delivery process of IPT-G demonstrated some benefits including: 1) Positive impacts on Individuals and community; 2) Improved Interpersonal relationship; 3) IPT-G reduces depressive symptoms; 4) Loss and grief therapy; 5) Barriers to IPT-G delivery by CHWs; and 6) Capacity building and skills development. (Please see page 7, paragraph 4, methods section) |
|  | Software | Coding was done manually by grouping similar quotations from the text. (Please see page 11, paragraph 1,methods section) |
|  | Participant checking | FGDs was conducted among adolescent mothers who had received IPT-G intervention and also in-depth interviews among the health care workers was done post-intervention. (Please see page 10, paragraph 1, methods section). |
|  | **Reporting** |  |
|  | Quotations presented | Quotations identified individual perceptions and experiences in support of the themes (Please see page 10, paragraph 3, methods section) |
|  | Data and findings consistent | Data presented and the findings was consistent as demonstrated even during its discussion. (Please see pages 10-23, results section and also check the discussion section from pages 24-28) |
|  | Clarity of major themes | Major themes were reported as findings We structured our findings highlighting acceptability and impact of IPT-G when delivered by lay workers into three domains towards enacting behavior change(Improved outcomes for adolescents, improved health system and community strengthening) after the intervention: i) Capability-building skills on IPT-G, ii) Motivation-competencies in IPT-G, and iii) Opportunity-provision of continuous supportive supervision.  (Please see page 12, last paragraph up to page 13 paragraph 1, findings section) |
|  | Clarity of minor themes | Most of the minor themes from the study was shared in discussion section.(Please see page 24 up to page 28, discussion section) |
